# Round the Hospitals

**Published:** 1921-04-30

**Authors:** 


### ROUND THE HOSPITALS.

It is satisfactory to learn on the authority of the General Nursing Council that considerable interest has been aroused in the Conference of matrons and nursing experts to discuss training, the syllabus and examinations, which is taking place as this issue of The Hospital reaches our readers. So keen is this interest, and so widespread the desire to be fully informed concerning the proceedings, that the Council has rescinded its order excluding the Press. We hope, therefore, to give in subsequent issues a full account of the proceedings at this Conference, which cannot fail to be a notable one in the history of nurse-training.

The Birmingham and Three Counties Centre of the College of Nursing is to be congratulated on the promised visit of Princess Mary to Birmingham to visit the Scenic Fair at Bingley Hall on June 6. This fair is being organised to establish the local College Centre on a self-supporting basis and to provide it with a club and lecture room in the city for trained nurses. It aims further at the endowment of scholarships for the nurses of Warwickshire, Worcestershire and Staffordshire. The scheme is an ambitious one, but if the success of similar efforts in Lancashire and Yorkshire is any criterion of what may be expected from Birmingham—and who can doubt that it is?—the raising of the whole of the money required is a foregone conclusion. Princess Mary has consented to the presentation to her of those matrons of hospitals in Birmingham and the Three Counties who are helping in the fair.

The question of the provision of adequate district nursing for insured persons has advanced a step further. The alternative methods of contribution by Approved Societies under Section 21 of the Insurance Act or out of surplus funds were discussed by the Executive Committee of the Central District Nursing Council at its meeting on April 18, and it was resolved to inform the Approved Societies having members in London that the Central Council is in a position to arrange for an adequate district nursing service for all insured persons resident in London, and to invite them to make a contribution by a subvention based on the number of members in a Society is considered preferable to any scheme requiring payment either by the member or by the Society in respect of the number of visits paid. The reply from the Approved Societies will be awaited with interest.

The outcry which has arisen over the dismissal of three probationers from the Isleworth Infirmary illustrates the mischief which may be occasioned with the best intentions by the interference of an outside society imperfectly acquainted with the facts. At the recent meeting of the Brentford Guardians, Miss Cumberbatch, the Chairman of the Hospital Committee, reduced the matter to its right proportions, as one of those little outbreaks of insubordination which are apt to arise, none can tell how, and produce more excitement than they are worth. One of the dismissed probationers outstayed her leave, disobeyed orders, assumed a "don't care" attitude towards the matron, and left the building without leave. The conduct of the second "was considered derogatory to the institution," and the third left the institution without leave. The Poor-law Officers' Union issued a manifesto which, to say the least of it, had no very happy effect. The Board, having heard the report of the Hospital Committee, passed a resolution, with three dissentients, approving its action.

Unless the sum of £27,000 can be raised for the Scottish Branch of Queen Victoria's Jubilee Institute for Nurses, it seems to be threatened with extinction. The annual expenditure has of late years exceeded the income by £5,000, investments have sadly depreciated, and subscriptions are falling off. In short, Scotland is faced with the national misfortune of losing this great organisation altogether. It has 306 affiliated associations, and commands the confidence both of the medical profession and the public. The Council report that the Jubilee Institute constitutes the most efficient and economical basis for the national nursing service so imperatively needed. Further development and enlargement, in co-operation with the Board of Health, would enable it to fill all the requirements of the domiciliary medical service under contemplation. The Jubilee Institute has in effect succeeded too well. It has opened up a domain too vast to be tilled entirely by voluntary effort.

The Lewisham Guardians have decided on complete reorganisation of the nursing in the infirmary, to commence as soon as the new accommodation for probationers is ready. Every probationer who replaces a staff nurse means, it is estimated, a saving of £35 a year in salary without loss in the efficiency of the hospital. It has been decided to appoint a sister-tutor at a salary of £120, who will instruct

**ROUND THE HOSPITALS—**(*continued*).

probationers in theoretical and practical nursing during the study year of eight months, and assist during the annual holidays of the matron and assistant matron. The examinations will be conducted by a matron of some recognised training-school, instead of, as heretofore, by the medical superintendent and the matron of the hospital. Prizes are to be instituted for the probationers who do best in their examinations. These alterations should raise this finely equipped infirmary to a worthy place among the nurse-training schools in the country. The syllabus of training recently issued by the General Nursing Council has had its share in influencing the guardians in their well-thought-out scheme.

The nurses at the Park Hospital Nurses' Home have been saved only by the mild winter from serious discomfort. The building contains 184 bedrooms, the floors are all of concrete, and the result is very chilly and comfortless, for though there are fireplaces there are no fires, and the radiators in the corridors are inadequate for the size of the building. At the recent meeting of the Metropolitan Asylums Board it was recommended that a new calorifier be installed with hot-water pipes through all the rooms at a cost of £1,500. Unfortunately, the work must be spread over three years, the floors being dealt with in consecutive summers to avoid too heavy calls on the time of the staff.

A certain Chicago Health Commissioner has discovered, so he says, how to train a nurse in eight weeks "provided she knows certain things to start with." The idea is to prepare women to be of service in epidemics and undertake cheap nursing in average homes; it is also hoped, by the aid of the 4,000 women already expeditiously turned into "nurses" after this method, to popularise preventive medicine. The pupils "average around forty years of age," and more than half of them are married or widowed. The Chicago Health Department has been fortunate, it must be admitted, in discovering so many women capable of acquiring "the fundamentals of nursing" in a couple of months.

The question of setting free members of the nursing staff, so that they may sleep at home on the eve of their weekly day off duty, has been again under discussion by the Derby Board of Guardians. A previous decision against granting leave of absence for the night was questioned by one of the Guardians, who declared it to be a gross injustice to the nurses. It transpired that nurses, though sometimes free at 4 p.m., commonly remained on duty till 8 p.m., and it was considered inadvisable to sanction their leaving the premises at that hour except on some special occasion. The boon of spending the night at home is one which nurses particularly value, and in many institutions—Charing Cross Hospital, for instance—the time-table is so arranged that this can be done habitually before the weekly leave. It is a question of good organisation.

By the courtesy of the respective Councils we reproduce in the present issue (see p. 87) the seals of the three General Nursing Councils. The design of the Scottish seal is simplicity itself, the cross of St. Andrew on a shield within a circle. The goddess Hygeia forms the centre of the other two, with roses and daffodils on either side in the case of the seal for England and Wales, and the shamrock on that for Ireland, which latter bears also the word "Eire," which is the Gaelic or Erse for Ireland.

